# The American Dental Association

**Published:** 1875-11

**Authors:** 


### SELECTED ARTICLES.

---

### ARTICLE III.

### *The American Dental Association—Fifteenth Annual Session.*

SECOND DAY—*Morning Session.*

The Association was called to order at the usual hour, the President, Dr. Dean, in the chair.

The subject of "Physiology as related to the Health of the Dentist," was still under discussion; Dr. John Allen's paper being the basis upon which the discussions proceeded.

Prof. Taft said that a few years ago most of the prominent workers in the profession were broken-down men. An improvement, however, is visible in the last few years. The confinement to which the dentist is subjected is bad, the position he occupies in operating is bad, the inhalations are injurious. We ought to study all these things which pertain to the health of the dentist. The work of the dentist is physically laborious; it does not require muscular energy to so great an extent as rail-mauling and using the sledge-hammer; but it requires the holding of a steady hand; a steady and persistent effort running through the whole day is necessitated. The attention must be riveted; and the mental strain is very great. The surgeon feels exhausted after a short operation only. The two efforts, physical and mental, when prolonged, induce very great exhaustion. Other occupations require only one of these two methods of application; some require physical effort only, others mental. We should strive to prevent this being carried to an extreme. The attention should be diverted; something should be chosen that will wholly draw off the attention. Let the change be great, and the employment chosen wholly diverse. Fresh air, rest, freedom from strain and stress, must be provided. The incompatibility between patient and operator, which was spoken of yesterday, tends to break down the dentist.

Dr. Morgan. Some time since this subject attracted attention in the Mississippi Valley Association, and he (the speaker) had offered a reward of two hundred dollars for an essay on the diseases of dentists and their remedies. Dentists are peculiarly subject to renal diseases, hemorrhoids, and chest diseases. Many avoid the strain upon the abdominal muscles by sitting while operating; and he is glad to know that this is so. A constant strain is more exhausting than a much more severe one for a short time only. Men who can raise forty or fifty pounds at arm's length with ease, will break down if required to continue to sustain only ten pounds. This, the chief cause of the dentist's breaking down, is the result of just such constant tension, which does not occur in other occupations. There is too little attention paid to ventilating our offices. If we could have sushine it would be better, but it is not often possible. He had once lived twelve years in the country on a little farm, but afterwards removed to the city, and in a short time was a broken-down man. He took the alarm and resumed a more active life, riding in summer from six to eight o'clock in the evening, and had been benefited by it.

Dr Barrett, (N. Y.) What is to be done with patients with whom we can establish no proper nervous relations? There are patients whom we dread to see come into our offices; others again we can labor over all day and not feel it. There is some mysterious vitality which we do not understand. Till we can, as it were, discover the difference between death and life, till we can discover some means of preventing this influence, we must go on and be robbed of vital force.

Prof. Taft refuses to operate for incompatible patients. No cultivation will bring about compatibility. These patients, however, will form proper relations with other operators, and he sends them to others. He had noticed that patients sometimes "feel first-rate," while the operator is wilted out. Sometimes quite the contrary is the case. If he suffers from one night's insomnia, he inquires closely into the cause, and usually succeeds in finding and removing it, so that he rarely misses the second night. Ordinarily, the trouble may be removed.

Prof. McQuillen. If every one should refuse to operate for the incompatibles, what would become of them? What would be thought of a physician or a surgeon who refused to attend a patient on account of incompatibility? It is a confession of weakness to talk this way. There is often more incompatibility on the part of the practitioner than of the patient; exacting of the latter unflinching patience and endurance under the most painful operations without any manifestation of sympathy, and, when the slightest shrinking or resistance is made, giving by word and action unmistakable evidences of temper. No man can expect to control others until he has obtained the mastery over himself. Rarey conquered a horse that had been pronounced untamable in an incredibly short time by exerting his peculiar influence over him. We should control our patients not by brute force, but by kindness; making such an impression on them that they will feel there is a magnetic power they must submit to. It is through the heart that the incompatibilities can be reached; a kind word, a sympathetic look, and a pleasant manner will enable a patient to submit to the most painful operations at the hands of a man of feeling, who would not tolerate the touch of another who lacked that essential quality. The operator, in fact, should have that command over himself which will enable him to control the most refractory patients and at the same time manifest a gentleness of manner which will support and encourage the most timid, or he is not entirely fitted for the practice of the profession.

If anæsthesia is but privity of oxygen, as maintained yesterday, how is it that nitrous oxide and chloroform act so promptly that the effects are manifested almost instantaneously, while pearl-divers can remain under water deprived of air other than that in their lungs for two or three minutes? We do not expel all the air from the lungs in expiration or thoroughly inflate them in inspiration. The quantity that is uniformly changed in ordinary respiration is named *breathing air.* What can be drawn in over and above this in the deepest inspiration is called *complemental air.* After ordinary expiration, such as that which expels the *breathing air*, a certain quantity of air remains in the lungs which may be expelled by a more forcible and deeper expiration; this is termed the *reserve air.* A certain quantity still remains over which there is no voluntary control, and this is called the *residual air.* When the lungs have been once inflated, no force can remove all the air from the cells. The hydrostatic test for infanticide is founded off this principle. Cutting off the air, as in drowning and hanging, will undoubtedly produce insensibility or narcosis, with the possibility of resuscitation, but this cannot be regarded as evidence that anæsthesia is not due to the agent employed acting as a poison directly upon the nerve-centers, rather than by an arrest of the oxygenation of the nervous mass.

He had known from personal experience what insomnia or wakefulness meant. In former years, under the pressure of varied duties as a practitioner, teacher and editor, in order to get through the work that crowded upon him he had been compelled to sit up late at night and rise early in the morning, and not unfrequently the entire night had been passed in his library hard at work. To do this he had used coffee and wrapped wet towels around his head, to drive off the tendency to drowsiness. After a time sleep, which had thus been driven away, could not be obtained when it was sought for. And night after night was passed without it, and even when it came it was accompanied by dreams, in which the mind was actively engaged in the labors of the day, with a tenfold exaggeration of work and attendant annoyances and difficulties.

Insomnia is due to an engorged condition of the blood-vessels of the brain, and this can only be corrected by restoring the normal equilibrium of the circulation in every portion of the organism. Exercise in the open air, walking, riding, swimming, and particularly a current bath at Niagara (in which you take hold of a stout rope with both hands, and then the body vibrates upon the surface of the water like a flag in a stiff breeze,) will send the blood tingling to the extremities, and enable one to sleep soundly. This is far better than to have recourse to the use of drugs. Bromide of potassium has, however, been strongly recommended by Dr. Hammond for insomnia. He says it acts like a charm, diminishing the amount of blood in the brain, and allaying any excitement of the nervous system that may be present in the sthenic form of insomnia.

Dr. John Allen is glad to see that this subject is being discussed, and desires to see a spirit of inquiry manifested. The proportion of deaths by insanity in other professions is far less than in ours, as he has ascertained by inquiry, and there must be some cause for it, which we must find out. We must study nature's laws, and we can't amend or alter them, we must conform to them as nearly as possible. Temporary relief may be obtained from stimulants, but just so far as these elevate the system, so far it falls below in their absence. It takes more and more, and moderation cannot be commanded.

Prof. Shepard has not observed the tendency to insanity among dentists to any such extent as described in Dr. Allen's paper. There are many old men in the profession; one in Boston seventy-five years of age is in active practice. It may be admitted that this class of men do not do as good work as younger men; that is impossible; but they are well men. Many men in mercantile pursuits are taken to the asylums from perplexities of business, but he cannot recall an instance of insanity among dentists. A large majority of them are well men comparatively. The reason that so many of the professions are young men is, that thirty years ago our numbers were small. It is a question whether the practice is so depressing upon life-force. Over work will bring its legitimate result, and those who have fallen within his knowledge have done so from overwork. One esteemed brother, deceased just previous to our last meeting, worked till 3 A. M. in investigating, and carried on a large practice; and on the morning of his death was at work upon an article for this association. The great secret of keeping health is the enjoyment of it, and of one's business; and a man who does not enjoy his profession and go to it with pleasure in the morning, cannot accomplish the best results with the least expenditure. It is the high motive, the idea that one is doing good, that will sustain; the further from the idea of money one can get, the greater pleasure he will find in his work, and the greater immunity from declining health.

Dr. Barrett thinks there is a greater drain upon dentists than upon other professions. It is not the refractory patients that are most exhausting, but those that settle down and draw and draw till we sink down exhausted. No dentist could keep this up as long as the physician can his exertions.

Prof. Flagg is in sympathy with Dr. Barrett. We must make an effort to control patients. He had often diminished the magnetic current by interposing folds of napkins between himself and the patient. It is the contact of skin with skin that does the mischief. Disagrees with Prof. Shepard; there are old men in the profession, but they are easy-going ones; are there any delvers seventy-five years old and in the harness? There are perhaps exceptions, but they are the Von Humboldts. Our calling produces more drain in every direction than any other; it is the constant effort that breaks down, and the love of it does it more than antipathy. Agrees with Prof. O. W. Holmes that it were better for mankind if all the drugs (as they are used, or rather abused) were in the bottom of the sea, and all the worse for the fishes! Protests against the use of the bromide of potassium. It is a treacherous drug,—very good for a few times, but "plays out," and leaves the patient in a deplorable condition. Assafœtida is about as good as any drug for both patient and operator; it is safe and may be used continuously. They sugar-coat pills to make them pleasant, and we must sugar coat our operations. Mingle gentleness with firmness.

Dr. Allen's paper is admirable, and takes irrefutable ground. We overtax ourselves, not as business men do, in the inordinate exertion to make money, but in the legitimate practice of our profession. We break down in an average time of eight years. Rest and a change of occupation are what are needed.

The difference between sleep and anæsthesia is subtle. Sleep is not recuperative; unconsciousness is another thing. In sleep there is turgescence of the brain, but in unconsciousness there is an anæmic condition. When a man dreams of things he bas been doing during the day, he had better stop. When we wake up from sleep we feel refreshed, but there is no refreshment from anæsthesia. There is always danger in this condition. The speaker is a strong believer in anæsthesia; it is one of the greatest boons ever given to suffering humanity, and we gave it to the medical profession, which we are all the time helping to the best of our ability. It is a boon, but, like other powerful agents may be and is misused. As in the use of arsenic, it is said that only a little is used; gentlemen say they never give it to unconsciousness; but it must be understood that when a patient is only partially under its influence, every nerve is fully aroused, and the pain is more severe than ever. Believes that the nervous mass is affected and the sooner it is done the better it is. The first sense lost in anæsthesia is that of smell ; and it progresses in regular order. When the pons varolii is affected it is a most critical condition,—one next to death. It is not wonderful that when patients have been thrown upon the bridge of helplessness, and then operated upon and tortured there should follow headaches, insomnia, and hysterics. The daughters of leading medical men are in the same condition.

Dr. Atkinson. All that has been said in regard to nutrition has referred to the blood. We fail to see that these are misnomers. The polarization and depolarization are not immediately affected by the breathing of oxygen. He was delighted with the manner in which Dr. Flagg dealt with the subject. We deal with concomitants and not with causes. We have no right to say that we know anything about causes. Anæsthesia and sleep are different,—one is normal and the other is abnormal. When all the oxygen is taken out of the system it is dead. Any agent may be food, poison, or remedy according to the tension of polarity. Do not throw the remedies to the fishes, but throw away our misapprehensions about remedies. We must not take extreme views. What is temperament? Only a polarization of currents. We must oppose kindness to unkindness, be master of the situation, and do good, irrespective of money. We must control our patients; not by the Rarey method, however, for we can't always choke them down.

Prof. Taft wished to correct a possible misapprehension ; he did not mean irritability when he said incompatibility in regard to patients. The sluggish patients who manifest no opposition are often the worst. They are repulsive to the operator, and they have the advantage of us, for if the operator was repulsive to them, they would seek some other one. An irritable, fussy patient may be brought down if we have the power to do it, but the other thing is a different thing. Do not think you can dominate all patients; you will be exhausted and broken down in proportion as you do it.

The subject was then passed.

The Executive Committee made a report, recommending that the afternoons be devoted to clinics, which was adopted.

The report of the Publication Committee was read, and the committee were instructed to publish the Transactions for the present year.

The subject of "Pathology and Surgery" was postponed, and "Histology and Microscopy" was taken up.

Dr. Taft read a report written by Dr. W. H. H. Jackson, of Ann Arbor.

The writer stated that in making microscopical investigation of hypertrophied cement, he had been struck by the frequency with which atrophy occurs in connection with it. There are constant changes in the intensity of the two vital forces which build up and tear down the tissues. In a normal state these forces balance each other, but if either gains the ascendency, we have hypertrophy, or atrophy. Through these alternations we are able to prove that the osseous system renews its tissues,—though not so rapidly as the soft parts do. Teeth are often found to have changed from soft and tender, to hard and strong. The lamination of hypertrophied cement shows that there are periods of activity and cessation, as well as places of greater activity of action. Wave-lines show the extent of these actions.

In investigating, the writer, had noticed that the bone-corpuscles were most numerous at the beginning of each stage of activity, and at the points of most rapid growth. What function do these corpuscles fulfill? Are they necessary to the structure? and if so, why are they sometimes absent? Or are they results of pathological conditions, or masses of periosteal tissue enveloped in the rapidly developed cement?

The writer is inclined to the latter position. Too little attention is paid to the minute anatomy of the teeth; attention to it would save injuries and pain. We should work with great caution in excavating the incisors, as filaments of the nerve often extend far into the dentine.

The transverse grooves found on teeth have two distinct characters,—those with rounded edges being formed during the development of the teeth, either as a pathological result or from varying intensity of action of the formative process. The second class with hollow edges is the result of chemical action of the secretions of the mouth.

Prof. Stricker, of Vienna, has been making some interesting observations on the pathology of suppuration. He concludes that pus-corpuscles are formed by the division of the cells of the injured or diseased tissue.

Dr. Taft then read an additional report written by Dr. E. D. Swain, of Chicago.

The committee reported a decided barrenness during the year,—quite in contrast with the few years preceding. Certain observations conducted by Prof. H. S. Chase, the results of which had appeared in the *Missouri Journal*, were alluded to. The committee had examined Prof. Chase's specimens for themselves, in company with Prof. H. A. Johnson and J. W. Danforth, of Chicago, both histologists of high repute. They found that they presented the appearances shown in the drawings, though not quite so markedly. The bead-like appearances were well defined, and the specimens numerous. All the teeth were taken from persons under seventeen, before complete de velopment had taken place. Prof. Chase advanced the theory that the tubuli had been formed by a linear arrangement of the cells, the proximal ends being absorbed, this process continued forming tubuli. He also claims that the nucleus still remains in the calcified cell, retaining vitality which enables it under proper conditions to renew its action, and that through this vitality the changes which take place in dentine occur,—such as absorption of dentine from beneath a perfect gold filling uniting the pulp-cavity and cavity of decay. Prof. Chase claims that calcification and decalcification take place in teeth, and that the animal portion may be removed without chemical action.

Though the appearances are doubtless present, yet they do not establish the theory; it cannot be considered proved till Prof. Chase shall demonstrate the presence of the nuclei.

Mr. John Tomes has called attention to the appearances observed by Dr. Chase. In his work published in 1848, he describes the linear arrangement of the cells, their divisions and elongation, and the ultimate absorption of the proximal ends, thus forming a continuous tube; the intertubular spaces becoming filled with earthy matter, dentine is formed. The baccated appearance he ascribes to the preserved convexity of the cells, and a constriction at the point of junction.

It will therefore be seen that Prof. Chase differs from Mr. Tomes only as to the disposition of the cell-nuclei. His theories are somewhat different from the generally adopted ones, and stimulate scientific men to the most thorough investigation.

The report then described a very interesting case of a dentigerous cyst. A young lady, aged twenty-three, had her teeth extracted. The deciduous right cuspid was still present. After its extraction, the operator (Dr. Morse, of Richmond, Kentucky,) observed an enlarged condition of the alveolus, and, supposing that the permanent cuspid was in the jaw or that the root of the temporary remained, applied his alveolar forceps and cut through. Examining the mass in the beak of the forceps, he found that he had extracted *sixteen* miniature cuspid teeth. The floor of the cyst revealed the sockets of several of these small teeth. The patient informed Dr. Morse that the permanent right cuspid had erupted as a "tush," and had been extracted some years previous by her physician.

The committee had submitted these teeth to microscopical examination, and the results were embodied in certain photographs accompanying the report. They were found to be perfectly developed teeth as to enamel, dentine, cementum, and pulp-cavity.

The report then proceeded to consider the probable mode of development of these teeth, and arrived at the conclusion that the primary tooth-germ by gemmation produced sixteen daughter cells, each of which became a tooth.

Adjourned.

*Evening Session.*

The subject of microscopy was still under discussion.

Prof. McQuillen said that he was glad to know that gentlemen were undertaking researches themselves. There is too much disposition in this country to rely upon the observations of Europeans. The microscope is too much of a toy with many. Micro-photographs are very fair, but cannot take the place of the microscope. Nutrition is not a subject for microscopical observation; it is too subtle, and we can observe its stages only. He had once honestly believed the doctrine of "*omne vivum ex ovo*," and taught it for years,—but is now a skeptic in regard to it. He is not prepared to say that the doctrine is false, but does not feel assured that it is true. We have had apparent beginnings of life from a formless fluid. If we should insist on the necessity of a germ to form a crystal, we should be laughed at by most; but the doctrine is advocated by some. If it is not true, it is reasonable that out of a formless fluid life is evolved *de novo*. The existence of the vegetable is dependent upon its obtaining fluids and gases from the inorganic kingdom. We live upon dead matter; the somatic and molecular life of our food substances is destroyed by cooking. When does it pass from dead to living matter? when assimilation takes place or before? In former times Owen regarded the process of formation in dentine as a centripetal calcification of the pulp. We recognize a series of cells arranged in linear form, which undergo a change, and become odontoblasts, having prolongations between which calcification takes place, forming intertubular tissue. We want the results of original investigations, but after a man has worked hard all day he is unfitted for such duties, and for that reason we have very little of it. Our institutions should be endowed, and so situated that young men of ability may confine themselves to original investigations, and then America may make its contributions to science as do England and Germany.

Dr. Atkinson. It is a pity that the best men know so little. We should be thankful that anybody knows anything. We deal with histology as we would if we should view an audience

*en masse*, and attempt to describe it. We shall never know anything about life until we go to the bottom of the matter of function. There is a substratum denominated *atom*, which is the least manifestation of life that we know of. Atoms are endowed with life,—they can't be killed. We have been told that the molecular life of our food is killed. A statement like that is either a *lapsus linguæ*, or it shows an utter misapprehension of the subject. A soms coalesce and manufacture molecules; plasma is an aggregation of molecules. Something must die that something else may live throughout the range of organic life. We have crystalline life, and below that granular life, molecular life, and atomic life. A crystal is regularly arranged granules that are regularly arranged molecules that are regularly arranged atoms.

The doctrine of inorganic or azoic existence will not do in this day. If we wish to know the origin of life, we must define the territory we are speaking of; when the conscious life, has left the body, we have organic, cellular, and molecular life left, and that is the food which we delight to suck from the beefsteak. It is simply a polarization and depolarization of atoms that constitutes molecular mass. We cannot disrupt molecules without reducing to ultimate atoms; there is no such thing as death. Matter means mother. Every one who has followed me knows I have given as complete an answer to the question we are discussing, as that two and two are four. When the blessed love of the Father of light comes in and illumines us, we are endowed with the capability to perceive. The doctrine that "*omne vivum ex ovo*" is pretty old, as old as Harvey. If the protoplasmic mass is an egg, that is true; there are no bricks without mud,—there is no loaf without dough. What do Bastian's investigations prove? Only that these points are so small as not to have been detected before; they do not prove that the germs are not essentially eggs. What is an atom? It is in size about the two-hundred-millionth of an inch. If one side of it is warm and the other cold, there is polarization and depolarization, and that is crystallization. When we have investigated deep enough, we shall be prepared to understand the processes, and they will be as plain as the freezing of water.

The subject was then passed.—*Dental Cosmos.*

TO BE CONTINUED.

